# Acute Ramus Intermedius Occlusion Presenting as the South African Flag Sign on Electrocardiogram

**DOI:** 10.7759/cureus.93032

**Published:** 2025-09-23

**Authors:** Dya P Andryan, Raisa A Manan, Cynthia Parameswari, Robin Wibowo, Aprilianasary Utami Dewi

**Affiliations:** 1 Cardiology, Umar Wirahadikusumah Regional General Hospital, Sumedang, IDN; 2 General Medicine, Umar Wirahadikusumah Regional General Hospital, Sumedang, IDN; 3 General Medicine, Universitas Padjadjaran, Sumedang, IDN; 4 Interventional Cardiology, Umar Wirahadikusumah Regional General Hospital, Sumedang, IDN

**Keywords:** coronary artery variants, high lateral stemi, occlusive myocardial infarction, ramus intermedius, south african flag sign

## Abstract

Occlusion of the ramus intermedius (RI) artery is an uncommon but clinically significant cause of acute coronary syndrome, often mimicking other coronary occlusions and complicating diagnosis. We report the case of a 65-year-old man presenting with progressive typical angina chest pain whose electrocardiogram (ECG) showed the "South African flag sign", a pattern typically attributed to the first diagonal branch (D1) or left circumflex artery (LCx) occlusion. Coronary angiography, however, revealed a total occlusion with grade V thrombus of the proximal RI rather than D1 or LCx, and the patient underwent successful primary percutaneous coronary intervention (PCI) of the RI and left anterior descending artery. This case underlines that occlusive myocardial infarction (OMI) patterns such as the "South African flag sign" are not exclusive to D1 or LCx involvement but may also result from RI occlusion, emphasizing the need for clinicians to recognize atypical OMI electrocardiographic patterns for accurate diagnosis and timely reperfusion therapy. In addition, the ST-segment elevation in V5-V6 can serve as a clue to differentiate RI occlusion from D1, since the RI is located more laterally compared to D1 and its occlusion would result in ST-segment elevation in the lateral leads such as V5-V6.

## Introduction

The ramus intermedius (RI) artery is an anatomic variant that arises from the left main coronary artery between the left anterior descending artery (LAD) and the left circumflex artery (LCx). When present, the RI often functions as an additional diagonal-like or obtuse marginal-like branch, supplying the high lateral free wall of the left ventricle. Because of this unique distribution, ischemia or infarction in the RI territory may overlap with areas classically attributed to the first diagonal branch (D1) or LCx occlusion, thereby creating diagnostic uncertainty when interpreting electrocardiogram (ECG) findings. Moreover, due to overlap in perfusion territories, the ECG manifestations of RI occlusion may mimic those of LAD diagonal or LCx lesions, which complicates the attribution of culprit lesions based solely on ECG criteria [1-3].

Electrocardiographic markers of occlusive myocardial infarction (OMI) have gained increasing recognition [4]. One such marker, the "South African flag sign," typically reflects occlusion of the D1 or LCx and is associated with anterolateral or high lateral wall infarction [5-8]. However, the specificity of this pattern is not absolute, and its occurrence in less typical culprit lesions is rarely reported [9].

We present the case of acute RI occlusion manifesting with the South African flag sign on ECG. This report aims to highlight the importance of recognizing atypical OMI patterns, as the timely identification of non-classical culprit lesions is essential for guiding management and improving clinical outcomes.

## Case presentation

A 65-year-old man with no documented history of coronary artery disease presented to the emergency department with a three-hour history of severe, typical angina chest pain radiating to the left arm. On arrival, his vitals were as follows: blood pressure 128/78 mmHg, heart rate 86 beats per minute, respiratory rate 20 breaths per minute, oxygen saturation 97% on room air, and body temperature 36.7°C. Physical examination was unremarkable, with no signs of heart failure or hemodynamic instability.

**Figure 1:**
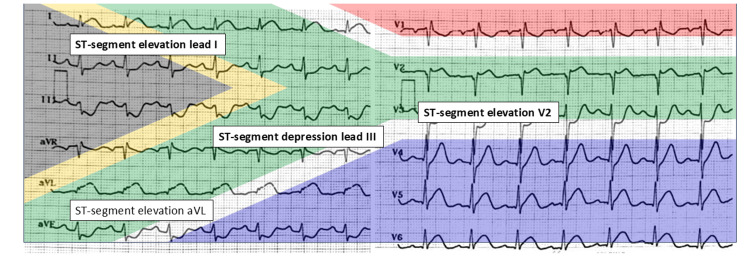
ECG of the patient showing the South African flag pattern The ECG pattern demonstrates ST-segment elevation in leads I, aVL, and V2, with reciprocal ST-segment depression in leads III and aVF, a finding commonly referred to as the "South African flag sign" when visualized in a landscape orientation. ECG: electrocardiogram; aVL: augmented vector left; aVF: augmented vector foot

The initial 12-lead ECG revealed slight ST-segment elevation in leads I, augmented vector left (aVL), and V2, along with reciprocal ST-segment depression in leads III and augmented vector foot (aVF), producing the so-called "South African flag sign" (Figure 1). High-sensitivity troponin I was elevated at 76.8 ng/L. The complete laboratory results are presented in Table 1.

**Table 1:** Lab values of the patient

Lab parameter	Value	Reference
Hemoglobin	12.1 g/dL	14-17.5 g/dL
Leukocyte	10,920/mm^3^	4,500-10,000/mm^3^
Thrombocyte	346,000/mm^3^	150,000-450,000/mm^3^
Hematocrit	40%	40-52%
Random blood sugar	96 mg/dL	100-150 mg/dL
Creatinine	0.7 mg/dL	0.5-1.1 mg/dL
Sodium	135 mmol/L	135-148 mmol/L
Potassium	3.5 mmol/L	3.5-5.1 mmol/L
Calcium	8.5 mmol/L	8.1-10.4 mmol/L
High-sensitivity troponin I	76.8 ng/L	<19 ng/L

**Figure 2:**
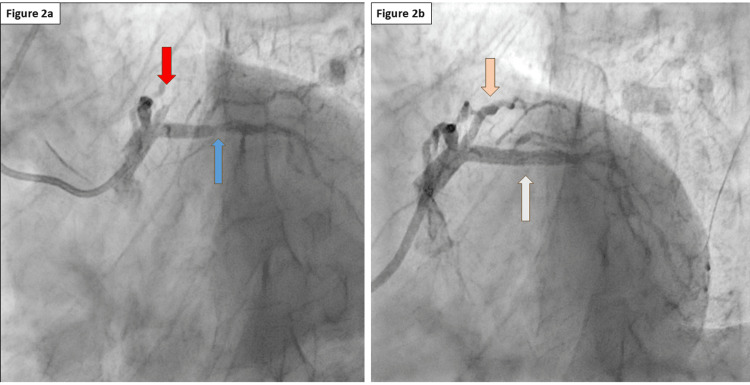
Coronary angiography images (a) Total occlusion of the RI (red arrow) and critical stenosis of the LAD (blue arrow). (b) The RI (orange arrow) and LAD (white arrow) following early PCI. LAD: left anterior descending artery; RI: ramus intermedius; PCI: percutaneous coronary intervention

Urgent coronary angiography demonstrated a total occlusion, thrombolysis in myocardial infarction (TIMI) 0 flow, with grade V thrombus in the proximal RI artery-mid-region (Figure 2a, red arrow). There was 90% stenosis of the proximal LAD with no significant disease in the right coronary artery (Figure 2a, blue arrow) and non-obstructive atherosclerotic changes in the LCx. Primary percutaneous coronary intervention (PCI) was performed successfully with balloon angioplasty and stent placement in the RI (Figure 2b, orange arrow), followed by PCI of the proximal LAD (Figure 2b, white arrow) due to concomitant critical stenosis. Post-procedural TIMI 3 flow was achieved. The patient's hospital course was uneventful. He was discharged on aspirin 100 mg OD, ticagrelor 90 mg BID, atorvastatin 40 mg OD, bisoprolol 5 mg OD, and ramipril 5 mg OD.

## Discussion

The RI artery is an anatomical variant arising from the left main coronary artery and present in approximately 15-30% of patients undergoing coronary angiography, supplying high anterior and anterolateral segments of the left ventricle [1-3]. Zhang et al. classified the RI into three anatomical types: the first located adjacent to the interventricular groove (LAD group), the second positioned in the mid-region (middle group), and the third situated near the atrioventricular groove (LCx group) [10]. Variations in the coronary distribution of the RI can delineate the specific myocardial wall regions affected by ischemia in the event of an RI occlusion [11,12]. While occlusion of the RI is relatively uncommon compared to the other coronary arteries, its clinical significance is considerable because it can result in acute coronary syndromes with atypical electrocardiographic findings.

**Figure 3:**
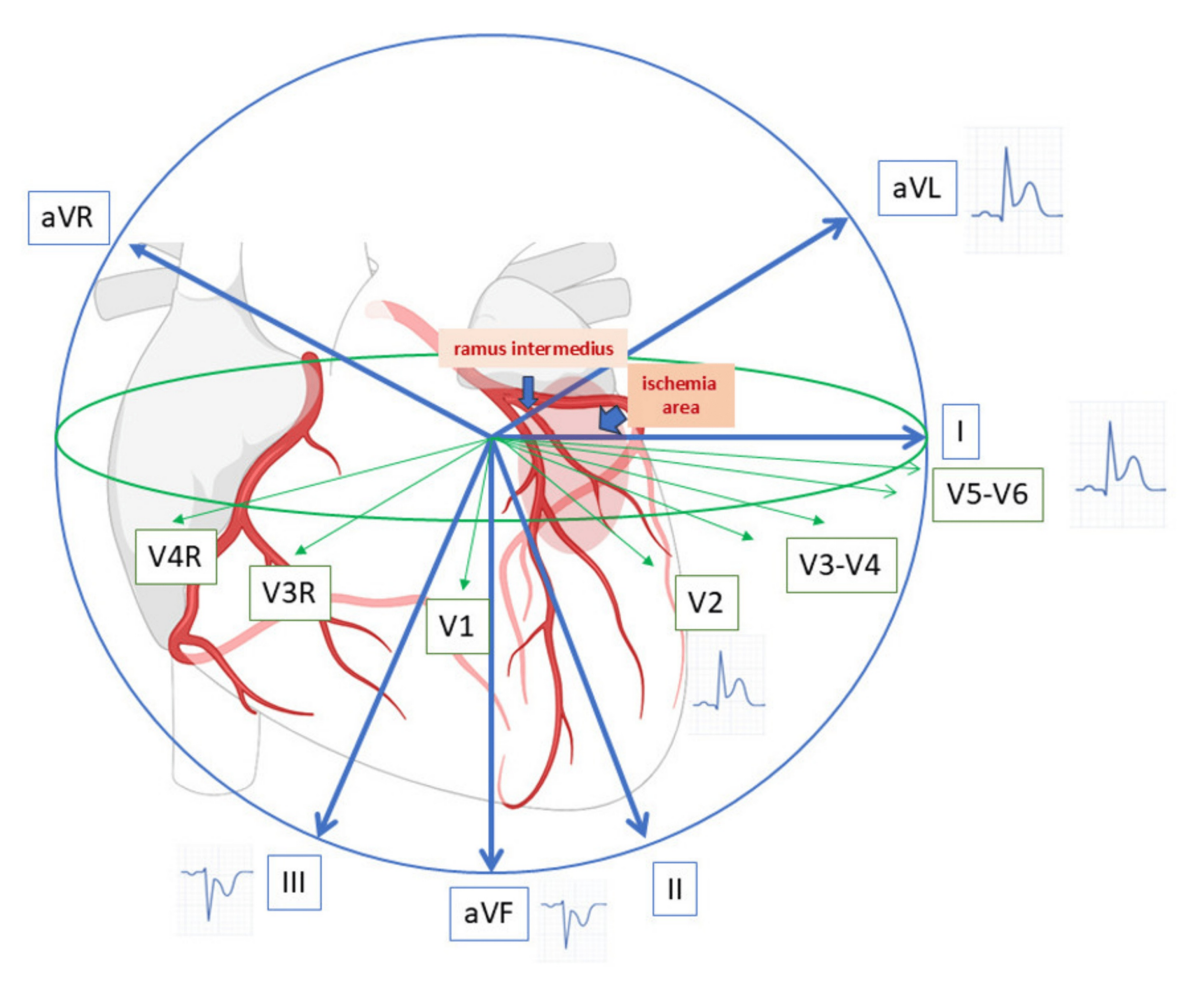
Vector illustration of ECG in RI occlusion The South African flag pattern, typically caused by D1 occlusion, results in the ST-segment vector being directed toward leads I, aVL, and V2 while pointing away from lead III. Since the RI supplies a myocardial territory similar to that of D1, occlusion of the RI may produce comparable ECG patterns and ischemic areas. RI: ramus intermedius; D1: first diagonal branch; ECG: electrocardiogram; aVL: augmented vector left; aVF: augmented vector foot; aVR: augmented vector right

The "South African flag sign" is an ECG pattern characterized by ST-segment elevation in leads I, aVL, and V2, with reciprocal ST-segment depression in lead III, sometimes also with other inferior leads, and sometimes in V1 or V3. The term was introduced to describe a visual resemblance to the South African flag when ECG was documented in traditional 4×3 format, as seen in Figure 2 [5,8]. This pattern has traditionally been associated with occlusion of the D1 or the LCx, both of which also supply the high lateral myocardial territory. Since the RI supplies a myocardial territory similar to that of D1, occlusion of the RI may produce comparable ECG patterns and ischemic areas [13]. As the RI is positioned slightly more lateral than D1, in our case, leads V5-V6 also demonstrate slight ST-segment elevation, corresponding to the vector orientation aligned with lead I (see Figure 3). We believe the low troponin level in the setting of a total occlusion is due to the sample being taken within the first hour of care, combined with the relatively small myocardial supply area.

Our case demonstrates that the South African flag sign, while commonly linked to D1, rather than identifying a specific culprit artery, more accurately reflects a particular ischemic territory [9,13]. In this patient, the culprit lesion was located in the proximal RI, underscoring the importance of considering RI as a potential source of high lateral infarction when this ECG pattern is encountered. This case adds to the limited literature describing RI occlusion as a cause of the South African flag sign and reinforces the need for clinicians to broaden their differential when confronted with high lateral OMI patterns. Prompt recognition of these atypical ECG findings can facilitate early intervention and improve outcomes in acute coronary syndromes.

## Conclusions

The South African flag sign is traditionally regarded as a marker of D1 branch occlusion, reflecting ischemia of the anterolateral or high lateral myocardial territory. However, this case demonstrates that the same electrocardiographic pattern may also occur in the setting of RI artery occlusion, owing to the overlap in coronary supply distribution between these vessels. The presence of RI, an anatomic variant originating between the LAD and LCx, expands the potential differential diagnosis when interpreting high lateral OMI patterns. ST-segment elevation in V5-V6 may help distinguish RI occlusion from D1, as the RI's more lateral position means its occlusion is more likely to produce elevation in the lateral leads.

Recognition of coronary anatomical variants and their corresponding ECG manifestations is therefore essential for clinicians evaluating patients with acute chest pain. Misattribution of culprit lesions may lead to diagnostic delays or incomplete revascularization strategies. By broadening awareness of atypical presentations, particularly those involving less common vessels such as the RI, physicians can improve the accuracy of early OMI diagnosis and ensure timely reperfusion therapy. Ultimately, integrating knowledge of coronary anatomy with the evolving OMI paradigm enhances clinical decision-making and contributes to better patient outcomes in acute coronary syndromes.

## References

[1] O'Brien JP, Srichai MB, Hecht EM, Kim DC, Jacobs JE (2007). Anatomy of the heart at multidetector CT: what the radiologist needs to know. Radiographics.

[2] Koşar P, Ergun E, Oztürk C, Koşar U (2009). Anatomic variations and anomalies of the coronary arteries: 64-slice CT angiographic appearance. Diagn Interv Radiol.

[3] Khachatryan A, Chow RT, Srivastava MC (2024). The ramus intermedius: a bridge to survival in the setting of triple-vessel total occlusion. Cureus.

[4] McLaren J, de Alencar JN, Aslanger EK, Meyers HP, Smith SW (2024). From ST-segment elevation MI to occlusion MI: the new paradigm shift in acute myocardial infarction. JACC Adv.

[5] Ricci F, Martini C, Scordo DM (2025). ECG patterns of occlusion myocardial infarction: a narrative review. Ann Emerg Med.

[6] Rajendran G, Mahalingam S, Kagne R, Nathan B (2021). The South African flag sign-an electrocardiographic sign to predict the culprit artery. QJM.

[7] Luckmann J, Win S, Ajagbe T, Samarawickrama T (2025). The South African flag sign: a key indicator of acute D1 occlusion. Cureus.

[8] Durant E, Singh A (2015). Acute first diagonal artery occlusion: a characteristic pattern of ST elevation in noncontiguous leads. Am J Emerg Med.

[9] Swarath S, Maharaj N, Hall A, Frederick JM, Seecheran R, Seecheran V, Seecheran NA (2023). The South African flag sign: an electrocardiographic flag for all coronary territories?. J Investig Med High Impact Case Rep.

[10] Zhang DQ, Xu YF, Dong YP, Yu SJ (2023). Coronary computed tomography angiography study on the relationship between the ramus intermedius and atherosclerosis in the bifurcation of the left main coronary artery. BMC Med Imaging.

[11] Abuchaim DC, Spera CA, Faraco DL, Ribas Filho JM, Malafaia O (2009). Coronary dominance patterns in the human heart investigated by corrosion casting [Article in Portuguese]. Rev Bras Cir Cardiovasc.

[12] Galbraith EM, McDaniel MC, Jeroudi AM, Kashlan OR, Suo J, Giddens D, Samady H (2010). Comparison of location of "culprit lesions" in left anterior descending coronary artery among patients with anterior wall ST-segment elevation myocardial infarction having ramus intermedius coronary arteries versus patients not having such arteries. Am J Cardiol.

[13] Monaghan M, Sreenivasan S (2020). A red flag ECG. Circulation.

